# Computer Simulation on the Cooperation of Functional Molecules during the Early Stages of Evolution

**DOI:** 10.1371/journal.pone.0035454

**Published:** 2012-04-13

**Authors:** Wentao Ma, Jiming Hu

**Affiliations:** 1 College of Life Sciences, Wuhan University, Wuhan, Hubei, China; 2 College of Chemistry and Molecular Sciences, Wuhan University, Wuhan, Hubei, China; Hungarian Academy of Sciences, Hungary

## Abstract

It is very likely that life began with some RNA (or RNA-like) molecules, self-replicating by base-pairing and exhibiting enzyme-like functions that favored the self-replication. Different functional molecules may have emerged by favoring their own self-replication at different aspects. Then, a direct route towards complexity/efficiency may have been through the coexistence/cooperation of these molecules. However, the likelihood of this route remains quite unclear, especially because the molecules would be competing for limited common resources. By computer simulation using a Monte-Carlo model (with “micro-resolution” at the level of nucleotides and membrane components), we show that the coexistence/cooperation of these molecules can occur naturally, both in a naked form and in a protocell form. The results of the computer simulation also lead to quite a few deductions concerning the environment and history in the scenario. First, a naked stage (with functional molecules catalyzing template-replication and metabolism) may have occurred early in evolution but required high concentration and limited dispersal of the system (e.g., on some mineral surface); the emergence of protocells enabled a “habitat-shift” into bulk water. Second, the protocell stage started with a substage of “pseudo-protocells”, with functional molecules catalyzing template-replication and metabolism, but still missing the function involved in the synthesis of membrane components, the emergence of which would lead to a subsequent “true-protocell” substage. Third, the initial unstable membrane, composed of prebiotically available fatty acids, should have been superseded quite early by a more stable membrane (e.g., composed of phospholipids, like modern cells). Additionally, the membrane-takeover probably occurred at the transition of the two substages of the protocells. The scenario described in the present study should correspond to an episode in early evolution, after the emergence of single “genes”, but before the appearance of a “chromosome” with linked genes.

## Introduction

According to the logic that “the simpler, the more possible to emerge from a non-life background”, life in the beginning should have been in some simple form (yet capable of Darwinian evolution). Hence, when it was revealed that RNA, acting as genetic material sometimes instead of DNA, could also act as functional (catalytic) molecules instead of proteins [1], [2], it began to be popularly believed that some early life forms were based solely on RNA, referred to as the “RNA world” [3]–[5]. An extreme version of this hypothesis states that the RNA-based life was just the earliest form (“RNA first”), re-emphasized by recent evidence concerning the plausibility of prebiotic nucleotide synthesis [6], [7]. Alternatively, the earliest life form may be based on some type of RNA-like polymer, in a pre-RNA world (“RNA later”) [4], [5]. For convenience, the present model was constructed and described in an “RNA first” view; however, similar conclusions may also be applicable for the molecular cooperation present in a pre-RNA world.

**Figure 1:**
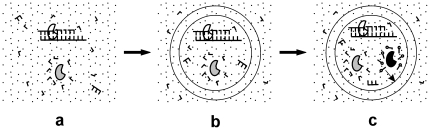
A scheme describing the cooperation of ribozymes in the early stages of the RNA world. Dots denote raw materials. “L-shapes” denote nucleotides. “Ball-sticks” denote amphiphiles. (**a**) RNA replicase (“Rep”, white) may cooperate with nucleotide synthetase ribozyme (“Nsr”, grey) in the “naked” stage. (**b**) Rep and Nsr may cooperate in the pseudo-protocell substage. (**c**) Rep, Nsr and amphiphile synthetase ribozyme (“Asr”, black) may cooperate in the true-protocell substage. The arrow shows that the amphiphiles may join the membrane of the protocell.

For the “RNA first” view, it has long been proposed that the first functional RNA to emerging was a ribozyme catalyzing the template-directed copying of RNA [4], [5], which may spread in a nucleotide pool by favoring its own replication (called “RNA replicase”, here “Rep” for short). We have shown this plausibility by computer simulation assuming that the Rep could adopt a simple ligase form [8]. Alternatively, some ribozyme(s) catalyzing the synthesis of nucleotides (“nucleotide synthetase ribozyme”, here “Nsr” for short), by supplying building blocks around itself and thus favoring its own replication, may also have emerged first; the plausibility of this has also been shown by our simulation work [9]. No matter which ribozyme was first, it is interesting to see whether the two different functional RNAs, self-replicating independently, could coexist in the same system while competing for a limited source of raw materials and, moreover, cooperate in this “naked” stage (Fig. 1a).

Evolution would eventually move into a protocell stage that included a membrane. The “cellular” coexistence/cooperation of Rep and Nsr is an interesting topic (Fig. 1b), especially in comparison with their cooperation in the naked stage. In the protocell stage, some ribozyme(s) synthesizing membrane components (“amphiphile synthetase ribozyme”, “Asr” for short) [10], [11] may have emerged, by contributing to the increase of the cellular space, thereby favoring the influx of additional raw materials and thus benefiting its own replication. The plausibility of this concept has been shown by our computer simulation work [12]. Protocells would then govern their own membrane synthesis, thereby becoming “true-protocells”. Consequently, it is quite interesting to incorporate the third ribozyme, i.e., Asr, into the system to determine whether it can coexist/cooperate with Rep and Nsr (Fig. 1c), and also to observe the behavior differences between “pseudo-protocells” (containing Rep and Nsr, but not Asr) and “true-protocells” (containing all three ribozymes).

The importance of the coexistence/cooperation of self-replicating molecules in early-life evolution was suggested quite early, even before the proposal of the RNA world hypothesis. In the early 1970s, Eigen [13] suggested that non-enzymatic template replication would have low fidelity and could only sustain information carried by short nucleic acids (<50 nt) transferring from generation to generation. Short self-replicating nucleic acids without (enzyme-like) functions would compete with each other, leading to the result that only the “fittest” species (in the sense of acting as a template) would survive. According to Eigen, the emergence of a larger genome would have to involve function-carrying molecules, i.e., proteinaceous enzymes. Therefore, he proposed that there should be some self-organizing system in early life, in which one of the short self-replicating nucleic acids, by its coded polypeptides, favored self-replication of the next, therefore finally forming a closed cycle, called a “hypercycle”. A key problem with this concept is how such a complicated system could emerge in the origin of life, especially when both transcription and translation machineries need to be considered.

Following the identification of ribozymes [1], [2] and the proposal of the RNA world hypothesis [3], it appeared that short self-replicating RNAs may act as function-carriers themselves. Consequently, the collective system proposed by Eigen could be replaced by a corresponding system purely based on RNA, which has a significantly reduced complexity and would be more likely to emerge in the origin of life. This is not the sole implication of the RNA world on Eigen's theory. If, as shown by computer simulation studies, short self-replicating RNAs could act as replicases (if adopting a simple ligase form, they may be shorter than 50 nt) [8] and could evolve towards higher efficiency and fidelity [14], the need to overcome Eigen's error threshold by the coexistence/cooperation of functional self-replicating species should no longer exist. However, the emergence of the molecular coexistence/cooperation remains important, because it appears to be the most natural path to complexity and efficiency after the emergence of individual functional RNAs.

After Eigen's work, some alternative mechanisms of coexistence/cooperation of functional self-replicating molecules (referred to as “replicators” following Dawkins [15]; just “RNA” in the view of the RNA world) were proposed. For example, the metabolic coupling model suggested that replicators catalyzing intermediate steps of monomer synthesis (i.e., Nsr-series) could coexist/cooperate [16]–[20], and the stochastic corrector model (SCM) emphasized the role of group selection of functional replicators within a protocell that was dependent on the protocell division [21]–[23]. The models approached reality and in some aspects are better than the hypercycle model (for a review, see [24]). However, these studies are still limited. In particular, only a little attention has been paid to the coexistence/cooperation of molecules with different (unrelated) functions (e.g., it was suggested that Rep may appear from parasites of the Nsr-series system [18]). Noticeably, the synthesis of membrane components, as an important function of the protocell, i.e., Asr, has never been considered. Additionally, there was negligible parallel exploration of the naked and protocell systems (a recent study [25] is an exception, but this study only focused on the interaction of Rep and parasites, without explicitly taking into consideration other functions).

In our previous simulation work, we have built explicit explanations describing the origin of individual functional RNA species, i.e., Rep [8], Nsr [9] and Asr [12]. The next stage in our study is the exploration (using similar models) of the plausibility of the coexistence/cooperation of these ribozymes with different (unrelated) functions, which represents a natural way towards complexity/efficiency in early evolution. In particular, as Rep functions in template-directed replication, Nsr functions in the metabolism of genetic and functional materials, and Asr is involved in the metabolism of the membrane, it can be concluded that the three functional RNA species included in the present study cover the fundamental requirements for the so-called minimal protocell [26]–[32]. Overall, the aim of this study was to show whether self-replicating RNA species, with functions at these different aspects, could coexist/cooperate (in both naked and protocell forms) while competing for common resources in the same system. The study also provides insights into the possible conditions and the history of this early evolution, after the advent of single genes yet before the emergence of chromosomes with linked genes.

## Methods

**Figure 2:**
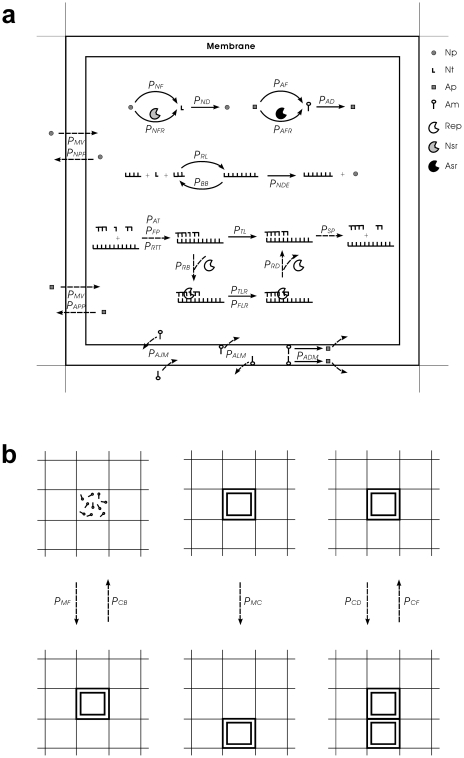
Events occurring in the model and their associated probabilities. Solid arrows represent chemical events and dashed arrows represent other events. (**a**) The events occurring in a grid room. Legends: Np, nucleotide precursor; Nt, nucleotide (A, U, C, or G); Ap, amphiphile precursor; Am, amphiphile. This version is for the true-protocell system. For the pseudo-protocell system, the events concerning Asr would not occur. For the naked system, the events concerning amphiphilic molecules and their precursors would not occur; there is no membrane at the edge of the grid room; nucleotides and RNA may also move to an adjacent grid room (see Table 1, note b). (**b**) Events concerning the behaviors of the protocells. When a protocell move to an adjacent (top, down, left, or right) naked grid room, the protocell would push away molecules in that room. When a protocell divides, amphiphiles on the membrane and molecules in the protocells would be distributed randomly between the two offspring protocells. One of the offspring protocells would occupy an adjacent naked grid room and push away molecules in that room.

The simulation is based on a Monte-Carlo model, in which each event in the system may occur with some numerical probability in a time (Monte-Carlo) step. The mechanism of the model is the same as our previous work studying the behaviors of Rep [8], Nsr [9] and Asr [12]. An *N*×*N* grid was used for the system, with toroidal topology to avoid edge effects. Only molecules within the same “grid room” could interact. A “grid room” represents a square in the grid. It is referred to as a “grid cell” in traditional stochastic cellular automaton. Here, to avoid its confusion with protocells in the model and to emphasize it as a space for molecules to reside, we call it a “grid room”. In a time step, each event may occur with some probability, as explained below (also see Fig. 2).

A nucleotide precursor may transform to a nucleotide (randomly as A, U, C, or G) with the probability *P_NF_* (see Table 1 for a description of the abbreviation and those appearing in the following). A nucleotide may decay into a nucleotide precursor with *P_ND_*. A nucleotide residue at the end of an RNA chain may decay with *P_NDE_*. A nucleotide may be ligated with another nucleotide or an RNA chain at its end with *P_RL_*. A phosphodiester bond within an RNA chain may break with *P_BB_* (the probability is multiplied by *F_BO_* for RNA out of the protocells). An RNA may turn into a template (unfolding) with *P_RTT_* and attract nucleotides or oligomers with *P_AT_* by base-pairing (the probability of false base-pairing at each residue site is *P_FP_*; for an oligomer, the base-pairing test is applied for all its residues). Nucleotides and oligomers aligned adjacently on the RNA template may be ligated to each other with *P_TL_* (template-directed ligation). The substrates or the products on the RNA template can dissociate if base pairs between them can separate (each base pair may separate with *P_SP_*).

**Table 1:** Parameters used in the Monte Carlo simulation.

Probabilities	Descriptions	Values	
		Fig. 3 c	Fig. 5 d
*P_AD_*	Amphiphile decaying into its precursor (out of membrane)	0.01	0.01
*P_ADM_*	Amphiphile decaying into its precursor within membrane	1×10^−4^	1×10^−4^
*P_AF_*	Amphiphile forming from its precursor (not catalyzed)	1×10^−3^	1×10^−3^
*P_AFR_*	Amphiphile forming from its precursor (catalyzed by Asr)	0.9	0.9
*P_AJM_*	Amphiphile joining membrane	0.9	0.9
*P_ALM_* a	Amphiphile leaving membrane	1×10^−4^	1×10^−4^
*P_APP_* b	Amphiphile precursor permeating membrane	0.1	0.1
*P_AT_*	RNA attracting nucleotides/oligomers by base-pairing	0.2	0.3
*P_BB_*	Phosphodiester bond breaking in an RNA chain	1×10^−6^	1×10^−6^
*P_CB_*	Protocell breaking	1×10^−5^	1×10^−5^
*P_CD_* b	Protocell dividing	0.1	0.5
*P_CF_*	Protocell fusing	5×10^−4^	1×10^−4^
*P_FLR_*	Ligating with false base-pairing on template (by Rep)	0.1	0.1
*P_FP_*	False base-pairing in RNA attracting nucleotides/oligomers	0.01	0.01
*P_MC_*	Movement of a protocell	0.01	0.05
*P_MF_* b	Membrane forming	0.1	0.1
*P_MV_* b	Movement of a nucleotide, amphiphile or their precursors	0.2	0.5
*P_ND_*	Nucleotide decaying into its precursor	0.01	0.01
*P_NDE_*	Nucleotide decaying into its precursor at RNA's chain end	1×10^−4^	5×10^−5^
*P_NF_*	Nucleotide forming from its precursor (not catalyzed)	2×10^−4^	2×10^−4^
*P_NFR_*	Nucleotide forming from its precursor (catalyzed by Nsr)	0.9	0.9
*P_NPP_* b	Nucleotide precursor permeating membrane	0.01	0.1
*P_RB_*	Rep binding onto RNA template	0.9	0.9
*P_RD_*	Rep dropping from RNA template	0.9	0.9
*P_RL_*	Random end-to-end ligation of nucleotides and RNA	1×10^−6^	1×10^−6^
*P_RTT_*	RNA turning to a template (i.e., unfolding)	0.5	0.5
*P_SP_* b	Base-pair separating	0.5	0.5
*P_TL_*	Nonenzymatic template-directed ligation	2×10^−4^	2×10^−4^
*P_TLR_*	Template-directed ligation catalyzed by Rep	0.9	0.9

Others	Descriptions	Fig. 3	Fig. 5
*F_BO_*	Phosphodiester bond breaking out of protocells: *P_BB_*×*F_BO_*	10	5
*F_OP_* a	Factor for the effect of osmotic pressure	5	5
*F_SI_* b	Factor for the effect of Donnan's equilibrium	5	2
*L_AM_*	Lower limit of amphiphiles to form protocell membrane	500	300
*N*	The system surface is defined as an *N*×*N* grid.	40	40
*T_APB_*	Total amphiphile precursors introduced in the beginning	6×10^4^	4×10^4^
*T_NPB_*	Total nucleotide precursors introduced in the beginning	8×10^4^	8×10^4^

a The probability of the amphiphile leaving the membrane is assumed to be *P_ALM_*/[1+*F_OP_*×*n*/(*b*/2)^3/2^], where *n* is the quantity of inner impermeable ions, including nucleotides and RNA (measured by the number of nucleotide residues), and *b* is the quantity of amphiphiles within the membrane. Wherein, *b*/2 (there are two layers in the membrane) is a “scale” representation of the surface area of the membrane. Consequently, (*b*/2)^3/2^ is a scale representation of the cellular space. Thus, *n*/(*b*/2)^3/2^ is a representation of the concentration of the ions. *F_OP_*×*n*/(*b*/2)^3/2^ represents the consideration for the “osmotic pressure effect”; a higher concentration of the inner impermeable ions would cause the protocell to be more swollen, and thus amphiphiles within the membrane are less likely to leave [57].

b The probability of RNA moving is assumed to be *P_MV_*/*m* ^1/3^, where *m* is the mass of the RNA, relative to a nucleotide. The probability of the membrane forming is assumed to be 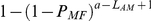, where *a* is the number of amphiphiles in the grid room. The probability of a nucleotide precursor permeating into a protocell is assumed to be [1−(1−*P_NPP_*)×(*L_AM_*/*b*)^3/2^]/[1+*F_SI_*×*n*/(*b*/2)^3/2^]; the probability of amphiphile precursors permeating into a protocell is assumed to be 1−(1−*P_APP_*)×(*L_AM_*/*b*)^3/2^; and the probability of the protocell dividing is assumed to be *P_CD_*×(1–2×*L_AM_*/*b*), where *n* and *b* have the same meanings as in note a. These assumptions are completely the same as the ones in Ref. [12], and are therefore not explained in full detail.

c This set of parameter values is for the cases in Fig. 3b and c (the protocell stage). Parameter values for the case in Fig. 3a (the naked stage) are the same except that *N* = 20, *P_MV_* = 5×10^−4^, *T_APB_* = 0, *F_BO_* = 1.

d This set of parameter values is the common parameter list for the parameter analysis in Fig. 5b and c (also in Fig. 6b and c; the protocell stage). The common parameter list for Fig. 5a (also for Fig. 6a; the naked stage) is the same except that *N* = 20, *P_MV_* = 1×10^−3^, *T_APB_* = 0, *F_BO_* = 1. The set is somewhat different (underlined) from that for the cases in Fig. 3 in order to show the influence of the parameters more clearly.

An amphiphile precursor may transform to an amphiphile with *P_AF_*. Amphiphiles (with a lower limit of quantity *L_AM_*) may assemble into membrane at the edge of a grid room with *P_MF_*, encompassing molecules within it and forming a “protocell”. A free amphiphile may decay into an amphiphile precursor with *P_AD_*, whereas one within a protocell membrane (not within the protocell, just on the membrane) may decay with *P_ADM_*. A free amphiphile may join a protocell's membrane with *P_AJM_*, whereas an amphiphile within a protocell's membrane may leave it with *P_ALM_*. Nucleotides and RNA are assumed to be impermeable, whereas a nucleotide precursor and an amphiphile precursor may diffuse across the membrane with *P_NPP_* and *P_APP_*, respectively. A protocell may divide into two with *P_CD_* and two adjacent protocells may fuse into one with *P_CF_*. A protocell may break (into free amphiphiles) with *P_CB_*.

An RNA containing a characteristic domain (presumed arbitrarily) may function as a corresponding ribozyme: Rep, Nsr, or Asr. However, if the length of the RNA equals or exceeds twice the characteristic domain, it is deemed that the “correct” structure would be interfered by the redundant residues and the RNA would not act as the ribozyme. A Rep molecule may bind onto an RNA template with *P_RB_* and drop from the template with *P_RD_*. If there is a Rep on the template, the template-directed ligation may occur with *P_TLR_*. However, if one or both base pairs flanking the ligation site are false, the ligation will not occur unless another probability, *P_FLR_*, is satisfied. In the model, *P_FP_* and *P_FLR_* are two parameters associated with the replication fidelity. The replication fidelity will increase when *P_FP_* and *P_FLR_* are small. An Nsr molecule may catalyze the formation of a nucleotide from a nucleotide precursor with *P_NFR_*, and an Asr molecule may catalyze the formation of an amphiphile from an amphiphile precursor with *P_AFR_*.

Before the next time step, molecules and protocells in a grid room may move into adjacent rooms. A nucleotide, a nucleotide precursor, an amphiphile, or an amphiphile precursor may move with *P_MV_*, whereas a protocell may move with *P_MC_*. The factors *F_OP_* and *F_SI_*, as well as detailed assumptions concerning some events mentioned above have been explained in the notes of Table 1.

Some considerations were taken into account to develop a logical setting of the numerical probabilities. Ribozymatic reactions should be much more efficient than corresponding non-enzymatic reactions, so *P_TLR_*>>*P_TL_*, *P_NFR_*>>*P_NF_*, and *P_AFR_*>>*P_AF_*. “Template-directed ligation” should be significantly more efficient than “random ligation”, so *P_TL_*>>*P_RL_*. Nucleotide residues in an RNA chain should be protected. Here, nucleotides within the chain are assumed to be unable to decay, whereas those at the end of the chain decay at a rate obviously lower than that of free nucleotides, i.e., *P_NDE_*<<*P_ND_*. Amphiphiles within membrane should be protected, so *P_ADM_*<<*P_AD_*. Owing to the self-assembly feature of amphiphilic molecules, *P_MF_*>>*P_CB_* and *P_AJM_*>>*P_ALM_*. The movement of molecules should be easier than protocells, so *P_MV_*>*P_MC_*. Other considerations may include: *P_BB_* may be at the same order as *P_RL_*, *P_ND_*>*P_NF_*, *P_AD_*>*P_AF_*, and *P_NPP_*≤*P_APP_*.

In a simulation case for the naked stage, nucleotide precursors in the quantity of *T_NPB_* were introduced in the initial step, and Rep and Nsr were inoculated at an early step. In a simulation case for the protocell stage, nucleotide precursors in the quantity of *T_NPB_* and amphiphile precursors in the quantity of *T_APB_* were introduced in the initial step, empty protocells were inoculated soon after the initial step, and then protocells containing ribozymes (Rep and Nsr for the pseudo-protocell substage; Rep, Nsr and Asr for the true-protocell substage) were inoculated at an early step. “Internal” events in the model, as described above, govern the whole dynamic process, step by step, occurring in the system.

## Results

### Molecular coexistence: naked versus cellular

The simulation showed that the functionally different ribozymes may spread together in the system, both in naked and protocell forms (Figs. 3 and 4). Here “spread” means that the molecule (copy) number increases after an initial inoculation and reach equilibrium later. Although in the model (see Methods) a ribozyme would be shorter than twice the characteristic domain (assumed here identical in length for different ribozymes), there is still the possibility that an RNA has different functions at the same time, if there is sequence-overlap. RNAs with two functions at the same time have been observed in our simulation; however, they are infrequent and cannot spread (data not shown in Fig. 3). The likely reason is that the dual-function ribozymes would be longer, and thus more difficult to fully replicate and more likely to degrade when compared with ribozymes with a single function.

**Figure 3:**
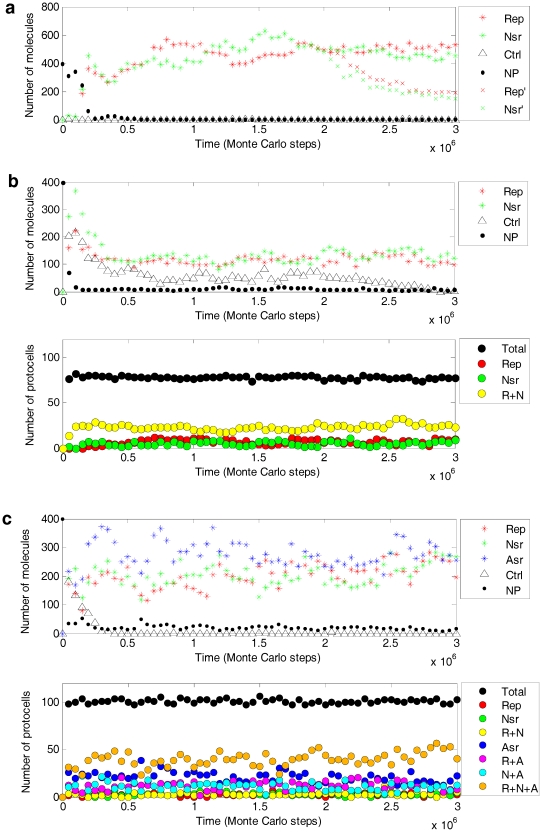
Co-spread of the ribozymes (the color-coded legends have been explained in detail in the following). The characteristic domain for a ribozyme or the control RNA species is a stem-loop “X_3_X_2_X_1_LLLLY_1_Y_2_Y_3_”, in which an “L” denotes a nucleotide in the loop, whereas the nucleotides in the stem, X_1_, X_2_, and X_3_ are complementary (by Watson-Crick pairs or a G-U pair) to Y_1_, Y_2_, and Y_3_, respectively. The loop nucleotides are “AGUC” for Rep (red stars), “ACUG” for Nsr (green stars), “AUCG” for Asr (blue stars), and “UCAG” for the control (triangles). Nucleotide precursors (dots) are represented in a 1/200 scale relative to the number of the ribozymes (e.g., 400 denotes 8×10^4^). The parameter values have been listed in Table 1. For the naked stage (**a**), 10 grid rooms, chosen randomly, were each inoculated with five molecules of Rep, Nsr and the control at step 1×10^4^ (cf. Fig. 4a); “x-shapes” show the tendency change of Rep (red) and Nsr (green) when *PMV* was enlarged to 0.01 (originally 5×10^−4^) at step 2×10^6^. For the pseudo-protocell substage (**b**) and the true-protocell substage (**c**), 10 grid rooms, chosen randomly, were each inoculated with an empty protocell at step 1×10^3^; ten grid rooms, chosen randomly, were each inoculated with an protocell containing five molecules of the ribozymes (Rep and Nsr in **b**, plus Asr in **c**) and the control at step 1×10^4^ (cf. Fig. 4b and c). Black circles represent total protocells. Other circles represent protocells containing Rep, Nsr and Asr (orange); Rep and Nsr but not Asr (yellow); Rep and Asr but not Nsr (magenta); Nsr and Asr but not Rep (cyan); only Rep (red); only Nsr (green); and only Asr (blue).

**Figure 4:**
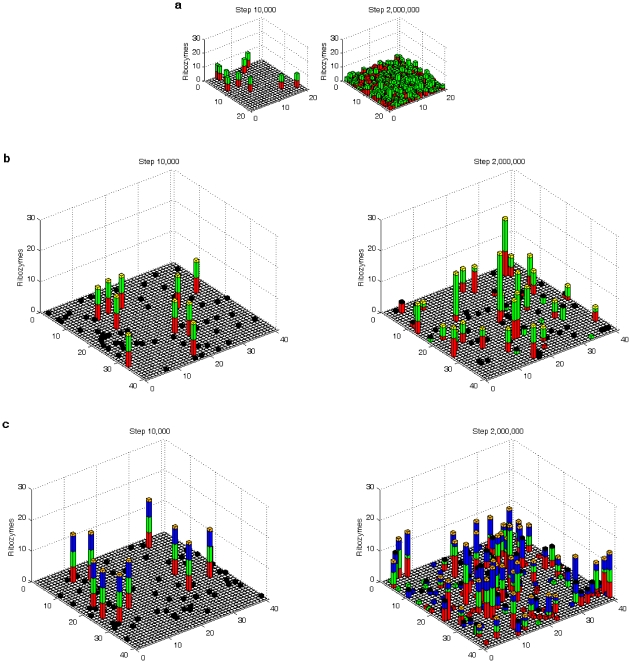
The spatial distribution of the ribozymes before and after their co-spread. The horizontal plane is the *N*×*N* grid. A bar in a grid room represents the number of ribozymes, Rep (red), Nsr (green) and Asr (blue), in a stacked form. If the ribozymes are actually within a protocell occupying the grid room, a “cap” (different colors with different meanings, see below) is added on the top of the bar. The cases are the same as the ones described in Fig. 3. Two snapshots, one at the step of the ribozyme-inoculation (step 1×10^4^) and another at a step after the ribozyme-spread (step 2×10^6^), were taken for each stage. For the naked stage (**a**), 10 grid rooms, chosen randomly, were each inoculated with five molecules of Rep, Nsr and the control RNA species (not drawn here) at step 1×10^4^. For the pseudo-protocell substage (**b**), 10 grid rooms, chosen randomly, were each inoculated with a protocell containing five molecules of Rep and Nsr and the control (not drawn here) at step 1×10^4^. A yellow “cap” denotes a protocell containing both Rep and Nsr, whereas a black cap denotes a protocell lacking at least one type of the two ribozymes. The empty protocells (without any ribozymes, represented only by a black cap), at this step, result from the spread of the 10 empty protocells inoculated at step 1×10^3^. The case for the true-protocell substage (**c**) is similar, except that Asr is inoculated together with Rep and Nsr, and an orange “cap” denotes a protocell containing all three ribozymes, whereas a black “cap” denotes a protocell lacking at least one type of the ribozymes.

The co-spread of the ribozymes may be sensitive to some parameters. For example, in the case shown in Fig. 3a (the naked form), when *P_MV_* was enlarged at an intermediate step, the previously co-spreading Rep and Nsr decreased immediately (x-shapes). This indicates that limited dispersal is important for the co-spread, similar to the results of our previous studies concerning the “individual” spread of Rep [8] and Nsr [9] in the naked stage.

**Figure 5:**
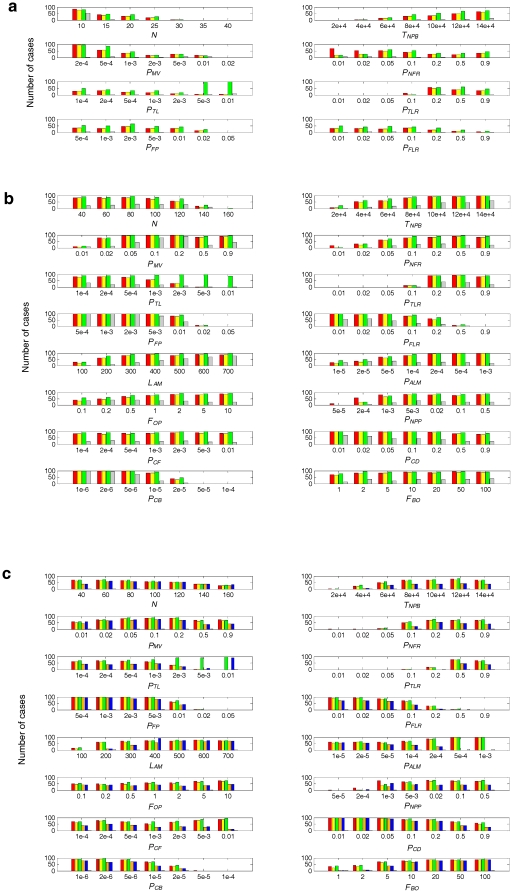
Influence of the parameters on the spreading chance of the ribozymes (part 1). The parameters included in this figure are tightly associated with the issues discussed in the paper, and the others are included in Fig. 6. A tick on a horizontal-axis denotes a value of the corresponding parameter. Random seeds 1–100 were used to initiate 100 different cases using such a parameter value, whereas values of the other parameters are set according to the common parameter list (see Table 1). The characteristic domains of the ribozymes and the control species, as well as the time step and the way of inoculating ribozymes (protocells), are the same as presented in Fig. 3. A bar is drawn with a height representing the number of the “spreading” cases, in which the corresponding ribozyme exceeds 50 (the number of initial inoculation) at step 5×10^5^. This criterion for the determination of a “spreading case” is adopted by experience to show the influence of the parameters clearly and also with consideration on the computational (time) cost. In a background of 100 cases, a bar can be seen as a representation of the “spreading chance” (in percent) of the corresponding RNA species: Rep (red), Nsr (green), Asr (blue) bars the control (grey). In addition, yellow bars represent the cases in which both Rep and Asr spread; orange bars represent the cases in which all Rep, Nsr and Asr spread. (**a**) The naked stage. (**b**) The pseudo-protocell substage. (**c**) The true-protocell substage. The parameters in the top half of **b** and **c** are parallel to those in **a**, whereas the parameters in the bottom half are those only affecting the protocell stage.

**Figure 6:**
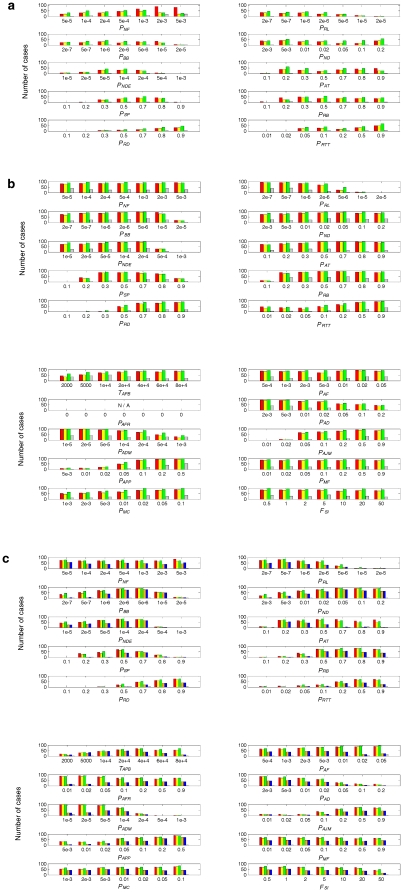
Influence of the parameters on the spreading chance of the ribozymes (part 2). The interpretation of this figure is the same as that of Fig. 5. (**a**) The naked stage. (**b**) The pseudo-protocell substage. (**c**) The true-protocell substage. The parameters in the top panels of **b** and **c** are parallel to those in **a**, whereas the parameters in the bottom panels of **b** and **c** are those only affecting the protocell stage.

To explore quantitatively the influence of the parameters, a systematic analysis was conducted, in which the effect of variation of a certain parameter on the “spreading chance” of the ribozymes was studied while the other parameters were fixed (Figs. 5 and 6). The parameter analysis confirmed that a small *P_MV_* is very important for the co-spread of the ribozymes in the naked stage (Fig. 5a, *P_MV_*). Additionally, the co-spread is also very sensitive to the dilution of the system. When *N* is larger (the grid becomes larger) and thus the concentration is lower (the total material in the system remains constant), the co-spread is not favored (Fig. 5a, *N*). However, for the protocell stage, the influence of these two parameters is much smaller (Fig. 5b and c, *P_MV_* and *N*). For example, for the naked stage, the spread of Rep and Nsr “disappears” completely when *N* was set to 35 (Fig. 5a), whereas for the pseudo-protocell substage, the spread of Rep and Nsr may occur even when *N* was set to 140 (Fig. 5b), and for the true-protocell substage the spread of Rep, Nsr and Asr continued even when *N* was set to 160 (Fig. 5c). Additionally, a too small *P_MV_* may be disadvantageous for the spread of the ribozymes in the protocell stage (Fig. 5b and c, *P_MV_*, from 0.05 to 0.01), which should be attributed to the weaker ability of the ribozymes to acquire raw materials elsewhere for their replication.

This obvious difference for the behavior of the naked and the cellular systems suggests that the corresponding two stages may have had significantly different environments in the origin of life. It has been shown that mineral surfaces may promote the random assembly of RNA from nucleotides [33], [34], protect RNA from degradation [35], [36], be compatible with the role of RNA as template [35]–[37] or as catalysts [38], and even aid in the formation of vesicles and the encapsulation of RNA into vesicles to form protocells [39]. It is possible that the naked stage may have taken place on some mineral surfaces, which, by absorption, provided an environment of high concentration [40] and low dispersal [41]. Alternatively, some eutectic phase in water-ice, which is advantageous for template-directed RNA synthesis [42], [43] and the stability of RNA chains [44], and even benefits ribozymatic reactions [45], [46], could also provide an environment of high concentration [40], [47] and low dispersal [45], [48]. Nonetheless, when protocells formed, the “habitat” may extend into bulk water, with an environment of low concentration and high dispersal. In the bulk water environment, a greater degradation rate of RNA out of protocells, because of the higher water chemical activity, would aid the accumulation of “genetic materials” inside the protocells, thereby strengthening the protocells' ability to “defend against” dilution (cf. Fig. 5b and c, *F_BO_*). Such a “habitat-shift” may provide these “RNA organisms” more opportunity to use raw materials or energy sources elsewhere.

Considering the obvious different environments that the naked and the protocell stages may have resided in, the simulation for the protocell stage (Fig. 3b and c, Fig. 4b and c) adopted a higher *P_MV_* and a larger *N* than that for the naked stage (Fig. 3a, Fig. 4a). However, all other parameters were identical for the simulation of the two stages, therefore facilitating a reasonable comparison between the two systems. The simulations of the pseudo-protocell substage and the true-protocell substage used the same parameter settings. The same strategy was also applied to the setting of the common parameter list in the parameter analysis (Figs. 5 and 6).

### About molecular cooperation

Noticeably, for the co-spread of the ribozymes in the protocell stage, in comparison with the pseudo-protocell system (Fig. 3b), the number of the ribozymes in the true-protocell system (Fig. 3c) reached a higher balance level after the spread. The phenomenon is common in our simulations, which should be attributed to the introduction of Asr. Asr catalyzes the synthesis of the membrane components and thus increases the cellular space, favoring the accumulation of raw materials for the synthesis of RNAs in the protocell [12], including Asr itself and the other two ribozymes. For the two systems, the total nucleotide precursors added at the beginning of the simulation (*T_NPB_*), as well as the other parameter values, was identical. Owing to the competition for the limited resources, it may be expected that the introduction of a third ribozyme would result in a drop in the balance level of the ribozymes. The opposite observation is a strong reflection of the cooperativity between the ribozymes.

The results of the parameter analysis also showed signs of cooperativity between the ribozymes:

1. For the naked stage, if non-enzymatic template-directed synthesis of RNA is efficient (Fig. 5a, *P_TL_*, 5×10^−3^ and 0.01), Nsr (green bar) was able to spread well without the spread of Rep (red bar). Noticeably, there is a sharp decrease for the spreading chance of Nsr, when *P_TL_* decreased from 5×10^−3^ to 2×10^−3^, which indicates that non-enzymatic template-directed synthesis could no longer support the replication of Nsr. An interesting point is that a further drop of *P_TL_* may increase the spreading chance of Nsr (Fig. 5a, *P_TL_*, from 2×10^−3^ to 2×10^−4^, green bar). The reason for this is that the decrease of *P_TL_* favors the spread of Rep (red bar) (because it has greater efficiency relative to the background non-enzymatic template-directed synthesis), and Rep would “aid” Nsr to in spreading chance. Similarly, Rep (red bar) may also help Asr (blue bar) to spread (Fig. 5c, *P_TL_*, from 5×10^−3^ to 1×10^−3^).
2. When Rep is not efficient and it cannot spread, Nsr or Asr may be suppressed too (Fig. 5a, b and c, *P_TLR_*, from 0.05 to 0.01).
3. When the fidelity of the template-directed copying increases because of a decrease in the ligation rate of false-paired substrates aligned on the template, which is a function of Rep, not only Rep but also the other ribozyme(s) have a better chance to spread (Fig. 5a, b and c, *P_FLR_*, from 0.9 to 0.1).
4. For the protocell stage, when the efficiency of Nsr decreased and its spreading chance decreased, the spreading chance of Rep (and Asr) was also observed to decrease (Fig. 5b and c, *P_NFR_*, from 0.1 to 0.01). The reason for this observation is that nucleotides are difficult to permeate through the membrane (they are assumed impermeable in the model, see the section “Membrane takeover” for an explanation) and RNA synthesis in protocells would rely on the nucleotide formation from nucleotide precursors permeating in from outside the cell. Consequently, Nsr is important.

### Membrane takeover

Modern cell membranes are mainly composed of phospholipids. However, fatty-acid membranes are more likely to have been the membrane for initial protocells, because fatty acids are simpler than phospholipids, and may have been readily synthesized in the prebiotic environment [49]–[51]. Then, when would phospholipid membranes have replaced fatty-acid membranes? Compared with fatty-acid membranes, phospholipid membranes are much more stable and less permeable [50], [51]; a phospholipid molecule has two hydrophobic chains, rather than the one chain found in a fatty acid molecule. It seems that phospholipid membranes were unsuitable before the emergence of membrane transporters, because it may be difficult for raw materials (nucleotides or their precursors) to permeate through a phospholipid membrane [50], [51]. Owing to the shortage of hydrophobic domains, RNA molecules are not good candidates to act as membrane transporters, though not impossible [50], [52]. If membrane transporters had to be made up of proteins, it seems that the membrane takeover would have been a very late evolutionary event, perhaps in an “RNA/protein world”.

However, detailed experimental data implied a different logic. The permeation is much more influenced by whether the raw materials are nucleotides or their precursors than by whether the membrane is a fatty-acid one or a phospholipid one. The permeation-balance time of nucleotides is usually on the order of hours or even days, either for the fatty-acid [53] or phospholipid membranes [54], whereas that of the nucleotide precursors, e.g., ribose, is only 1 or 2 minutes, either for the fatty-acid or phospholipid membranes [55]. Indeed, although it was shown in an experimental study that permeation of nucleotides across a fatty-acid membrane can support template-copying within the protocell, in the same study it was also shown that a membrane quite permeable to ribose may form a great barrier to nucleotides [56]. The molecular size and charge of the solutes should represent the determining factor. Considering that in the naked stage, Nsr may have already emerged [9] and been able to coexist/cooperate with Rep (as shown here), we can postulate that Nsr may have already existed in the initial protocells. In such a system, only the permeability of the nucleotide precursors is important. With respect to the permeability of nucleotide precursors, fatty-acid membranes would not be clearly superior to phospholipid membranes. Conversely, our analysis below suggests that phospholipid membranes would be clearly superior to fatty-acid membranes in some other aspects, which implies that the membrane takeover should have occurred quite early in evolution.

Considering their great difference in permeability, the nucleotides are assumed to be impermeable in the present model (for simplification), whereas nucleotide precursors may permeate with *P_NPP_*. It is not surprising to observe that the permeating rate of nucleotide precursors is a factor limiting the spread of the ribozymes (Fig. 5b and c, *P_NPP_*). However, it is clearly not the only factor (e.g., >10^−3^, *P_NPP_* is already not the limiting factor). It may be expected that the shortage of raw materials (see Fig. 3b and c, dots) is a key limiting factor, but notably, an increase of *T_NPB_* above a particular value may also have little influence on the spread (Fig. 5c, *T_NPB_*, from 8×10^4^ to 14×10^4^). The bottleneck may involve other factors. In the model, *P_FP_* (false base-pairing) and *P_FLR_* (“false” ligation catalyzed by Rep) are two parameters associated with the replication fidelity. When they are large, the error threshold [13] becomes a problem and the spreading chance of all the ribozymes would decrease (Fig. 5a, b and c, *P_FP_* and *P_FLR_*). For the protocell system, an increase of replicating fidelity, i.e., a decrease of *P_FP_* or *P_FLR_*, may push the spreading chance towards 100% (Fig. 5b and c, *P_FP_* and *P_FLR_*). However, replicating fidelity cannot be expected to be very high at this early stage, either for non-enzymatic template-copying or template-copying with a primitive Rep [5]. Here, we noticed two other factors: the probability of the protocell breaking (*P_CB_*) and the probability of the protocell dividing (*P_CD_*), the decrease of which would push the spreading chance of the ribozymes towards 100% (Fig. 5b and c, *P_CB_* and *P_CD_*). Interestingly, for these two factors, a phospholipid membrane would be superior to a fatty-acid membrane. First, the phospholipid membrane, being more stable, is less likely to break. Second, also owing to its stability, the phospholipid membrane would be noticeably more robust against division, which at this early stage may be caused by random physical forces in the environment as the protocell increases in size [49], [51].

It is easy to comprehend the importance of a small *P_CB_*. However, why is a small *P_CD_* also important? We note that a larger setting of the lower limit of the amphiphiles in a protocell membrane would favor the spread of the ribozymes (Fig. 5b and c, *L_AM_*), which indicates that larger cellular spaces would be advantageous. This can be explained by considering the random distribution of the “bagged genes” during the protocell-division. That is, “gene loss” in the offspring would occur more readily when the protocell is smaller and contains fewer copies of the genes (this idea was noted in the SCM model [21], [22]; however, their results and corresponding interpretation were somewhat different, which will be explained below in the section “Discussion”). The phenomenon of “gene loss” has been shown in the simulation cases here (protocells lacking at least one ribozyme: the bottom panel in Fig. 2b, red and green circles; the bottom panel in Fig. 2c, yellow, magenta, cyan, red, green and blue circles). Similarly, a smaller *P_CD_* would favor the spread of the ribozymes because the protocells would grow to a larger size before division. A larger cellular space would become more important when the types of genes in the “bag” increased and “gene loss” is more likely to occur.

Therefore, the phospholipid membrane may have taken over the fatty acid membrane quite early, before the prosperity of the RNA world and well before the emergence of proteins. Interestingly, some other results from the parameter analysis implied that the phospholipid membrane should have taken over the fatty acid membrane just accompanying the emergence of Asr, i.e., the emergence of true-protocells.

Experimental work on fatty-acid vesicles has indicated that the osmotic pressure brought about by increasing the genetic material within protocells would lead to a coupled growth of their membrane, because the tension in the membrane of the more swollen vesicles makes amphiphiles less likely to leave the membrane in the exchange processes of these membrane components between vesicles [57]. This core-membrane coupling, which does not involve any “biotic” function, should have characterized the pseudo-protocell substage. In the present model, *F_OP_* is a factor for this consideration (Table 1, note a), and the simulation result was in agreement with such a suggestion. When *F_OP_* is set larger, i.e., “the osmotic pressure effect” becomes greater, Rep and Nsr in the pseudo-protocells would have a greater spreading chance (Fig. 5b, *F_OP_*) because of the enhanced core-membrane coupling. Additionally, when the rate of amphiphiles leaving the membrane increases, Rep and Nsr also have a greater spreading chance (Fig. 5b, *P_ALM_*), because the exchange of membrane components becomes more intensive, thereby strengthening the osmotic pressure effect. This result suggests that the pseudo-protocell substage was likely to be dominated by fatty-acid vesicles, which have a less stable membrane and thus a higher rate of amphiphiles leaving the membrane [50], [51].

However, for the true-protocell substage, the core-membrane coupling would be triggered by membrane growth instead of the synthesis of genetic material in the protocell [12]. Asr, catalyzing the synthesis of membrane components, would contribute to the increase of the cellular space and favor the influx of more raw materials to synthesize genetic material. Apparently, the membrane of protocells with Asr would be more relaxed, and the tendency of amphiphiles leaving the membrane would be disadvantageous for Asr to implement its functional benefits. Consequently, Asr would have a less spreading chance as *P_ALM_* increases (Fig. 5c, *P_ALM_*). Additionally, the spreading chance of Asr would decrease when the rate of protocell fusion becomes higher (Fig. 5c, *P_CF_*), which is also a feature of protocells with unstable membranes. Therefore, phospholipid membranes, being more stable, would be more suitable for the true-protocells than fatty-acid membranes.

An additional reason why the fatty acid membrane is unlikely for the true-protocell substage comes from chemical consideration. The synthesis of fatty acids is difficult with respect to the formation of their C-C bonds. In modern cells, the biotic synthesis involves a rather complex process, and it is quite unlikely that simple ribozymes would accomplish this process successfully. In contrast, with prebiotically synthesized fatty acids and glycerol [51], the synthesis of glycerol esters and the subsequent phosphorylation to generate phosphatidic acid (the simplest phospholipid) appears to be much easier and would probably be within the capability of ribozymes [4], [58].

Therefore, we can imagine the scenario of the “membrane takeover”. The pseudo-protocell substage was governed by fatty-acid membranes. Subsequently, Asr emerged, catalyzing the synthesis of phospholipids (perhaps initially as glycerol diesters) that replaced the role of fatty acids. Interestingly, a recent experimental study [59] suggested that such membrane takeover may have occurred naturally because phospholipids joining into the fatty acid membrane would prevent the departure of fatty acids from the membrane, thus leading to vesicle growth at the cost of vesicles without phospholipids (in the exchange processes of membrane components between vesicles, similar to the osmotic pressure effect, as mentioned above). This would favor protocells containing Asr in the competition for a limited supply of lipids. Then the true-protocells, with a more stable membrane, would overwhelm the pseudo-protocells with fatty-acid membranes, and would govern the following stage in the evolution of protocells. Subsequently, accompanying the emergence of more and more additional types of functional ribozymes, the “bagged genes” mechanism would become more and more inappropriate, and a linked genome (chromosome) would be derived as a final solution to the problem of “gene loss” during protocell division [24], [60], thereby taking protocells more closer to modern cells.

## Discussion

The simulation was based on a model with a resolution at the level of monomers, i.e., individual nucleotides (in different types, A, U, G and C) and amphiphiles, and therefore should be very telling in tracing the fundamental rules governing these early life stages, which are characterized by the assembly and disassembly of genetic polymers and membranes.

**Figure 7:**
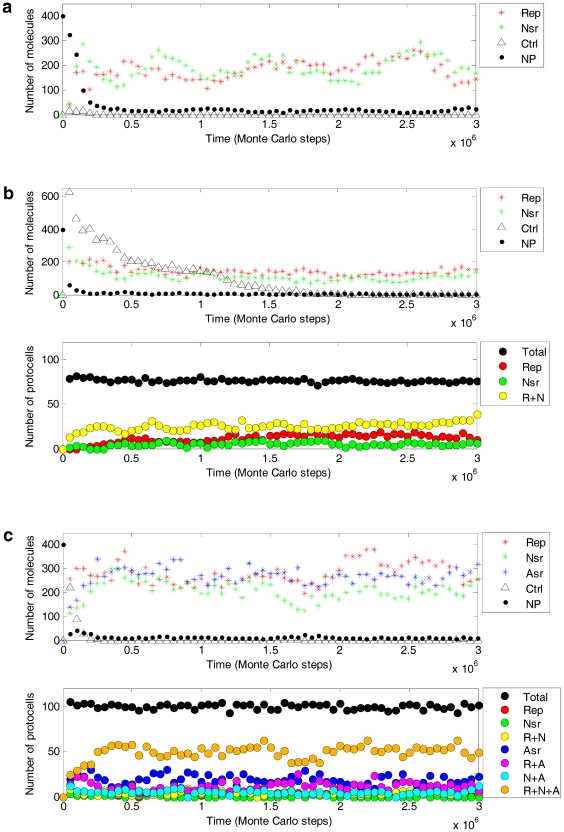
Co-spread of the ribozymes in the presence of selfish parasites. The interpretation of this figure is the same as Fig. 3. The parameter values are identical to those in Fig. 3, except that the control species (triangles) has a larger *P_RTT_* (0.9) than other RNAs (0.5), including the ribozymes, and therefore has a superior feature as template and is a selfish parasite.

For example, different from the replicator models [16]–[24], here template-directed synthesis of RNA is not a single event purposely assumed in the model but the outcome of collective behaviors of nucleotides and RNA nucleotide residues, which is based on the mechanism of base-pairing. As a parallel assumption, different functional RNA ribozymes are recognized according to their characteristic sequence domains. Therefore, all other sequences, short or long, derived from inaccurate copying (associated with *P_FP_* and *P_FLR_*), random ligation (associated with *P_RL_*) and degeneration of RNA chains (associated with *P_BB_*), would act as parasites in the model system. The co-spread of the functional ribozymes in the presence of these parasites provides a clearer picture of how robust the coexistence/cooperation of the ribozymes are against parasitization. Additionally, the control species inoculated together with the ribozymes (see triangles in Fig. 3) can be considered as parasites introduced *ad hoc*. Moreover, to make the results more convincing, we have done a simulation (Fig. 7) under the assumption that the control species has a superior feature acting as template (with larger *P_RTT_*), thus being a “selfish parasite”, like those in the replicator models. It was shown that there is no difference in quality (compared with Fig. 3), except that for the pseudo-protocell form, this selfish parasite may arrive at a rather high level in early steps of the simulation (see triangles in Fig. 7b). This may be attributed to the fact that the pseudo-protocells, without Asr, grow less efficiently and cannot divide in time to exclude the selfish parasite. However, the selfish parasite cannot destroy the co-spread of Rep and Nsr, and as time lapses, accompanying the division of the pseudo-protocells one generation after another, the selfish parasite decreases and finally becomes extinct.

Additionally, owing to the micro-resolution, some factors possibly influencing the results as discussed in previous studies, e.g., the product-inhibition in template-directed replication [61], [62] and the competition between ribozymes and their complementary chains [63], have been naturally included in the model.

Similarly, considering the behavior of the membranes of the protocells at the level of its components has provided valuable data. For example, it was revealed that a higher rate of amphiphiles leaving the membrane (*P_ALM_*) would disfavor the true-protocells (containing Asr), though favoring the pseudo-protocells (not containing Asr), which supports our hypothesis that the initial fatty-acid membrane should have been taken over by a more stable membrane (composed of phospholipids) when true-protocells appeared.

The present model, though not based on molecular dynamics, is very close to an *ab initio* model, considering its detailed description of the most fundamental events in the modeled system. With such a model, it is interesting to see that coexistence/cooperation of different functional RNA species (Rep, Nsr and Asr), self-replicating independently, could be so straightforward and robust, if only an RNA containing a characteristic domain (presumed arbitrarily) would function as a corresponding functional molecule. This suggests that such a scenario should have occurred naturally in the early evolution of life.

Notably, previous computer simulation research using replicator models reached some deductions relating to the ones in this report. While limited dispersal was found to be important for the spread of “replicators” functioning like Rep in the naked stage [14], some mixing was shown to favor the coexistence/cooperation of replicators with distinct functions in the synthesis of monomers of the replicators (Nsr-series) in the metabolic coupling model [18]. This seems reasonable because the synthesis of monomers would be easier to complete when all these replicators exist in a neighborhood. As an associated result, it was demonstrated that when replicators with function of replicase (Rep) appear in this system, limited dispersal is not important for the coexistence/cooperation of Rep and Nsr-series [18]. In the present model, Nsr is the only species facilitating the synthesis of nucleotides (i.e., it is not extended into the Nsr-series), so no completely parallel set of results are available. However, we showed here that for the coexistence/cooperation of Rep and Nsr, limited dispersal is very important (Fig. 3a; Fig. 5a, *P_MV_*), which at least appears puzzling when compared with their result. Where does the difference come from? In their model, the behaviors of the monomers were not described and the existence of all components of the Nsr-series in the neighborhood was seen as a manifestation of the availability of the monomers. A consequence of this approach was that the diffusion of monomers was not considered. The “dispersal” in their model was just the dispersal of replicators. In our model, the dispersal is a feature of the whole system, considering the movement of RNA, monomers and their precursors (see Table 1, the description of *P_MV_*, and note b). Since the diffusion of monomers should be more intensive than replicators, the omission is inappropriate. It could be expected that if the diffusion of monomers were considered in their model, their results would also show the importance of limited dispersal for the coexistence/cooperation of the Rep and Nsr-series. This is a sound example illustrating the importance of using a micro-resolution model, as described herein.

Another different result between the present study and previous work using replicator models involves the growth and division of protocells. In the SCM approach [21], [22], it is emphasized that different functional replicators with different replicating rates (in a sense as templates) could coexist/cooperate within protocells, because of group selection at the level of the protocells. That is, before the disappearance of the slower replicators, protocell division may occur, with random distribution (assortment) of the replicators between its offspring. Subsequently, the protocells with an appropriate proportion of functional replicators would be superior to the protocells short of the slower replicators. The group selection “corrects” the extinct tendency of slower replicators. The SCM implies that protocell division should occur in time before the disappearance of slower replicators, and the protocell could not grow large (see also [64], [65]). However, our results show that a lower rate of protocell division would favor the coexistence/cooperation of Rep, Nsr and Asr (Fig. 5c, *P_CD_*) by preventing “gene loss”, i.e., the protocell should grow large to contain more copies of each gene (ribozyme) before division, during which random distribution of the genes to its offspring would occur.

One reason for the difference may arise from the identical replicating rates of Rep, Nsr and Asr in our model with micro-resolution (because the events concerning replication are not different for the three ribozymes when they are acting as templates, see Fig. 2), unlike the replicating rates of different replicators in the SCM approach, which are different, assumed to follow Eigen's concept [13]. In the present model, the difference of Rep, Nsr and Asr is only concerning their functions, not their template features, because we have neither the knowledge nor reasons to define which ribozyme (and its complementary chain) should have a superior feature acting as a template. The competition here rests on the fact that all RNA species, either the ribozymes or the ones without any function (can be deemed as parasites), would exploit common raw materials (i.e., nucleotide precursors). A ribozyme would only benefit from its catalytic function, as the phenotype. In the pseudo-protocell system, the protocells containing both the Rep and Nsr (Fig. 3b, below, yellow circles) would be greater in number than the protocells containing only one of them; in the true-protocell system, the protocell containing all the Rep, Nsr and Asr (Fig. 3c, below, orange circles) would become the most prosperous. Apparently, the protocells containing both/all the ribozymes would be superior to other protocells. This shows a sense of “group selection”, but does not involve the “correcting” mechanism described in SCM; as mentioned above, a lower rate of protocell division would favor the co-spread of the ribozymes (to prevent “gene loss”). It was recently shown that if competition of replicators with different replicating rates (in a sense as templates) were subjugated, the “correcting” mechanism at the level of the protocell may no longer be significantly important [66]. Additionally, another possible reason may deserve attention. The SCM focuses on the coexistence/cooperation of species functioning as an Nsr series, whereas the present model focused on that of the species with unrelated different functions, i.e., Rep, Nsr and Asr. Species within the Nsr-series (catalyzing different steps in the synthesis of monomers) would be expected to be more sensitive to competition, because in front of their “common good” (to supply monomers for replication), they are equivalent.

**Figure 8:**
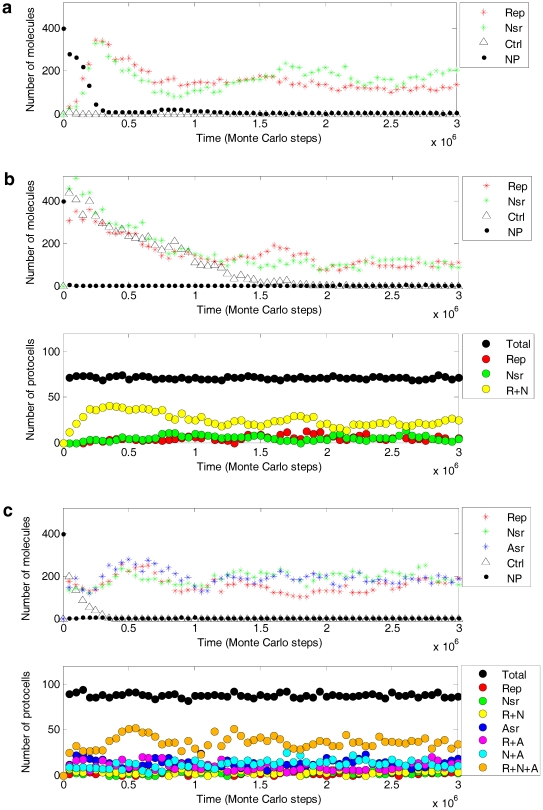
Co-spread of the ribozymes in a larger scale system. The characteristic domain for a ribozyme or the control RNA species is a stem-loop “X_8_∼X_1_LLLLY_1_∼Y_8_”, i.e., the domain has a length of 20 nucleotides. Nucleotide precursors (dots) are represented in a 1/400 scale relative to the number of the ribozymes (e.g., 400 denotes 1.6×10^5^). The manner of inoculating ribozymes (/protocells) and the figure symbols are the same as those used in Fig. 3. For the naked stage (**a**), *T_NPB_* = 1.6×10^5^, *P_NDE_* = 5×10^−5^, *P_AT_* = 0.3, *P_FP_* = 5×10^−3^, *P_FLR_* = 0.05, and the values of the other parameters are the same as in Fig. 3a. For the pseudo-protocell substage (**b**) and the true-protocell substage (**c**), *T_NPB_* = 1.6×10^5^, *L_AM_* = 600, *P_NDE_* = 5×10^−5^, *P_AT_* = 0.3, *P_FP_* = 5×10^−3^, *P_FLR_* = 0.05, *P_MV_* = 0.5, *P_ADM_* = 5×10^−5^, *P_ALM_* = 5×10^−5^, *P_NPP_* = 0.1, *P_APP_* = 0.2, *P_CB_* = 5×10^−6^, *P_CF_* = 1×10^−4^, *P_CD_* = 0.01, *P_MC_* = 0.05, *F_SI_* = 2, and the values of the other parameters are the same as in Fig. 3b and c.

The use of micro-resolution increases the complexity of the system, the model is some complex and therefore the simulation is computer-intensive. For simplification, the model adopts a two-dimensional grid system. In the system, a grid room can accommodate some molecules that are deemed as adjacent enough to interact with each other. Different from the stochastic cellular automaton (usually employed in the replicator models), in which one molecule occupies one grid room and interactions occur between neighboring grid rooms, this treatment would save computational costs and favor the simulation of larger system considering complicated interactions of a number of molecules, as in the present model. Regarding the possible initial habitats of the RNA world in the naked stage, though the modeled system is similar to a mineral surface [33], [35], [38] in the sense of its two-dimension feature, its topological structure aligns more closely to the eutectic phase in water-ice [42], [47], [48] or the “porous rock” suggested recently [19]. For the protocell stage, the membrane is assumed to assemble at the edge of a grid room, and thus the growth of protocells cannot be represented by the spatial organization per se (until protocell division). However, if a protocell can occupy more than one grid room, the model would become significantly more complicated. Hence, we preferred to overcome this issue by introducing some counterbalancing considerations into the model (Table 1, note a, concerning *P_ALM_*; note b, concerning *P_NPP_* and *P_APP_*). To avoid cumbersome computation, especially for the parameter analysis (Figs. 5 and 6), some parameters associated with the scale of the system were set smaller than the situation in reality, e.g., *T_NPB_*, *T_APB_*, *L_AM_* and the characteristic domain of the ribozymes (only as a simple stem-loop of 10 nt in length). However, single cases assuming a relatively larger system-scale are feasible, as shown in Fig. 8, in which the characteristic domain for the ribozymes (20 nt in length) already approaches that of small ribozymes found in nature.

Finally, although the deductions upon the evolving conditions or history in the origin of life (e.g., the importance of high concentration and limited dispersal for the naked stage; the schedule for the membrane takeover in the protocell stage) are interesting, the most impressive point in the present study should still be that based on a model describing the most fundamental events at the level of individual nucleotides and amphiphiles, the computer simulation demonstrates that different functional molecules beneficial to different aspects of self-replication can cooperate and spread in the same system, either in a naked form or in a protocell form. The coexistence and cooperation of these functional molecules represents a plausible path towards complexity and efficiency during the earliest stages of evolution. This is a conclusion with a higher sense, concerning the scientific aspect of the origin of life.
